# ABCA1-Labeled Exosomes in Serum Contain Higher MicroRNA-193b Levels in Alzheimer's Disease

**DOI:** 10.1155/2021/5450397

**Published:** 2021-03-08

**Authors:** Chen-Geng Liu, Yue Zhao, Yao Lu, Pei-Chang Wang

**Affiliations:** Clinical Laboratory of Xuanwu Hospital, Capital Medical University, Beijing 100053, China

## Abstract

**Objective:**

We aimed to establish a method to determine whether microRNA-193b (miR-193b) levels in ABCA1-labeled serum exosomes might serve as a marker for the diagnosis of Alzheimer's disease.

**Methods:**

We used immunocapture methods to determine the levels of ABCA1-labeled exosomal miR-193b in cultures of white blood cells (WBCs), red blood cells (RBCs), mouse hippocampal neuron HT-22 cells, and primary mouse neuronal cells. ABCA1-labeled exosomal miR-193b levels were also evaluated in the cerebrospinal fluid (CSF) and serum of APP/PS1 double-transgenic mice, as well as control subjects (*n* = 60) and study participants with subjective cognitive decline (SCD, *n* = 89), stage and mild cognitive impairment (MCI, *n* = 92), and dementia of the Alzheimer type (DAT, *n* = 92).

**Results:**

ABCA1 levels of exosomes harvested from the medium of HT-22 cells and neurons were significantly higher than those of RBCs and WBCs (*P* < 0.05). Exosomal ABCA1 from the CSF of APP/PS1 mice were transmitted to the serum of wild-type mice after injection, and high miR-193b levels were observed in both the serum and CSF after injection. The ABCA1-labeled exosomal miR-193b levels were higher in the CSF of MCI and DAT patients compared with the CSF of the control group (*P* < 0.05). The ABCA1-labeled exosomal miR-193b were also slightly higher (*P* > 0.05) in the serum of SCD patients and significantly higher in the serum of MCI and DAT patients compared with the serum of the control group (*P* < 0.05).

**Conclusion:**

This study provides a method to capture specific exosomes. Detection of serum exosomes labeled with ABCA1 may facilitate the early diagnosis of AD.

## 1. Introduction

Alzheimer's disease (AD) is a degenerative neurological disorder with progressive neuronal cell damage and apoptosis as the main cytological manifestations. The clinical manifestations include obstacles in progressive memory and language and cognitive dysfunction. With the acceleration of population aging, the absolute value of the number of AD patients has increased. The number of AD patients in China now exceeds 8 million, and the prevalence rate for people over 65 years old is 4-6% [1, 2]. Previous research suggests that pathophysiological changes associated with AD begin at least 10 to 25 years before dementia onset [3, 4]. The development of AD can be generally divided into subjective cognitive decline (SCD), mild cognitive impairment (MCI), and dementia of Alzheimer's type (DAT), the latter being the terminal stage. It has been confirmed that the brain of DAT patients has already suffered irreversible damage and that there is no effective treatment, so it is particularly important to detect and treat AD in the SCD and MCI phases, where the damage is relatively reversible and effective medical intervention is possible [1, 5–7].

Cerebrospinal fluid (CSF) tau protein and A*β* have been identified as biomarkers of AD; however, patients and their families often do not agree to CSF extraction for early diagnosis and screening of AD because of its invasive nature [6, 8]. Furthermore, the reality is that for patients with AD who have typical symptoms of DAT, there is currently no effective treatment. Therefore, the detection of CSF tau protein and A*β* is rare. With the continuous progress of AD biomarker research, the identification of noninvasive biomarkers for AD diagnosis is an emerging focus, especially for those with potential for early diagnosis [7, 8]. At present, research is centered on the value of small molecule nucleic acids and specific proteins in the diagnosis of AD; however, results have had little impact on AD diagnosis thus far, and more work is needed to develop noninvasive approaches to early diagnosis.

MicroRNAs (miRs) are a class of small (18-25 nucleotides), single-stranded noncoding RNAs involved in the posttranscriptional regulation of gene expression. In recent years, miRs have been shown to play important roles in several diseases, such as cancer, cardiovascular disease, and diabetes, as well as central nervous system diseases. Because of their stable character, altered miRs in tissues and organs may lead to the deregulation of miRs in body fluids such as cerebrospinal fluid (CSF), serum, and urine, either by cell destruction or secretion. Therefore, miRs are attractive targets in the search of novel biomarkers [9–11].

In a previous study, we reported that miR-193b downregulates amyloid precursor protein (APP) [11]. miR-193b is downregulated in hippocampal neurons of APP/PS1 transgenic mice, but total exosomal miR-193b is upregulated [11, 12]. This phenomenon suggests that a decrease of miR-193b in neurons may be correlated with the rise of secretory exosomal miR-193b. Further exosome protein profiling results showed that the expression of the ATP-binding cassette transporter A1 (ABCA1) in the CSF of AD patients is significantly higher than that of controls, suggesting that ABCA1 may be related to the increase of exosomal miR-193b. Due to the complex origin of CSF and peripheral blood exosomes, high-miR-193b exosomes may not be effectively isolated and detected, leading to false negative results in patients. To circumvent this issue, in the present study, we used the ABCA1 protein as a label to capture specific exosomes. Using this strategy, we assessed the value of ABCA1-labeled exosomal miR-193b in the diagnosis of AD, with implications for the early diagnosis of AD.

## 2. Methods

### 2.1. Study Population

A total of 333 subjects were randomly selected from the Xuanwu Hospital of Capital Medical University and the Beijing Xicheng District Guangwai Hospital from May 2018 to December 2019, of which 89 subjects were in the SCD group, 92 subjects were in the MCI group, and 92 subjects were in the DAT group. Another 30 randomly selected healthy subjects were recruited as the control group (Table 1). All patients or their legally authorized representatives provided written informed consent prior to sample collection. AD diagnoses were made by neurologists at Xuanwu Hospital and Guangwai Hospital, according to relevant diagnostic guidelines [13]. Subjects were excluded if they had a history of diseases of the nervous, endocrine, liver, kidney, cardiovascular, or cerebrovascular systems, and no subjects were recipients of lipid-lowering treatments. Venous blood was drawn in the morning, 12 hours after fasting. To avoid the influence of the exosomes released from blood cells, specimens were only allowed to stand for 30 minutes after hemagglutination. Blood samples were centrifuged at 3,000 g for 7 minutes (4°C) to separate the serum; the EDTA blood was centrifuged at 1,200 g for 7 minutes (4°C) to separate blood cells [11, 12]. The CSF was drawn within 2 h after the blood collection; the total cell amount of the CSF was less than 7.5 × 10^6^/l. Separated specimens were stored in liquid nitrogen before testing and were only allowed to freeze and thaw once. This study was reviewed and approved by the ethics committee of the Xuanwu Hospital of Capital Medical University and the Beijing Xicheng District Guangwai Hospital.

### 2.2. Blood Cell Separation and Culture

The red blood cells (RBCs) and white blood cells (WBCs) of SCD, MCI, DAT, and control groups were separated in a blood separation medium according to the manufacturer's protocol (Solarbio, Beijing, China). The separated RBCs and WBCs were washed in 37°C prewarmed phosphate-buffered saline (PBS) 5 times and then cultured in serum-free RPMI 1640 medium (Invitrogen, Carlsbad, USA) (1 × 10^7^/l) for 30 min, 1 h, 2 h, 4 h, and 6 h. The total miR-193b in the cells and medium as well as the exosomal miR-193b in medium were detected. The control, SCD, MCI, and DAT samples were run in triplicate.

### 2.3. APP/PS1 Double-Transgenic Mice

APP/PS1 double-transgenic mice in a C57BL/6J genetic background were purchased from the Institute of Laboratory Animal Science at the Chinese Academy of Medical Sciences & Comparative Medical Center. All animal protocols were approved by the ethics committees of the Xuanwu Hospital of Capital Medical University and the Beijing Xicheng District Guangwai Hospital. Matched nontransgenic mice were used as wild-type (WT) controls. At 3, 6, 9, and 12 months of age, CSF-like fluid was collected as previously described [11]. Briefly, the mice were sacrificed, and their brains were removed into a 35 mm dish. The cranial cavity and cerebral ventricles (lateral, third, and fourth ventricles) were rinsed with 500 *μ*l PBS, and CSF was harvested in PBS. The exosomes were then isolated using the method described below and were resuspended in 200 *μ*l PBS. Exosomes from 3-, 6-, 9-, and 12-month-old APP/PS1 double-transgenic mice and WT mice as well as a PBS control (100 *μ*l; *n* = 5 per group) were separately injected into the third ventricle of WT mice using a brain solid positioner (Stoelting, Illinois, USA). After 1 h, 2 h, 4 h, and 6 h, blood was taken from the eyeballs and was centrifuged at 3,000 g for 7 minutes to harvest serum immediately after blood coagulation. The CSF was also harvested at the same times after injection. The serum and CSF were used for exosome extraction and detection.

### 2.4. Cell Culture and Detection

The mouse hippocampal neuron cell line HT-22 (Cell Bank of the Chinese Academy of Sciences, Shanghai, China) was grown in antibiotic-free DMEM (Invitrogen, Carlsbad, USA) supplemented with 10% exosome-free fetal bovine serum (Umibio, Shanghai, China) at 37°C with 5% CO_2_. Sixteen-day pregnant WT and transgenic mice were sacrificed by CO_2_ inhalation, and then fetal primary mouse hippocampal neurons were isolated as previously described [11]. The isolated cells were seeded in 6-well plates coated with 10 mg/ml poly-D-lysine (Sigma, St. Louis, USA) in Neurobasal™ Media (Invitrogen) supplemented with 2% B27 supplement (Invitrogen), 2 mM glutamine (Invitrogen), 1 mM sodium pyruvate (Invitrogen), 5 mg/ml insulin (Sigma), and 40 mg/ml of gentamicin (Invitrogen) at 37°C with 5% CO_2_ [11–14]. The cells were cultured for 36 h for detection of ABCA1 protein, and the cell culture medium was also collected in parallel for the capture of ABCA1 protein-labeled exosomes and quantification of ABCA1 protein (*n* = 5 per group).

### 2.5. Isolation of Exosomes

The exosomes were isolated using the Total Exosome Isolation kit (Invitrogen) according to the manufacturer's instructions [12]. Briefly, serum, plasma, CSF, or medium was centrifuged at 2,000 g for 30 minutes to remove cells and debris. Next, 400 *μ*l of clarified sample was transferred to a new tube, and 0.4 volumes of the Total Exosome Isolation reagent was added. The serum/reagent solution was mixed and then incubated at 4°C for 30 minutes. The samples were centrifuged at 10,000 g for 10 minutes at room temperature, and then the supernatant was discarded and the pellet containing the exosomes was resuspended in 200 *μ*l PBS.

### 2.6. RNA Purification and miR Analysis

Total RNA in 100 *μ*l was purified by a spin column method using the miRNeasy Serum/Plasma Kit (QIAGEN, Hilden, Germany) according to the manufacturer's protocol [11, 12]. Total RNA yields were ~20 ng/ml and ~50 ng/ml in CSF and plasma, respectively, as assessed by the Quant-iT RiboGreen RNA reagent (Invitrogen). Total RNA in blood cells was extracted by a spin column method using the miRNeasy Kit (QIAGEN). miRs were reverse transcribed into cDNA using the miScript II RT Kit (QIAGEN) with 10 *μ*l reaction volume, followed by the TaqMan qPCR (QIAGEN) according to the manufacturer's protocol. Briefly, a 10 *μ*l PCR reaction contained 1 *μ*l cDNA, 300 nM TaqMan probe, 300 nM sense primer, and 300 nM antisense primer. Cycling parameters were 95°C for 10 min, followed by 40 cycles of 95°C for 15 s and 60°C for 1 min (Roche Light Cycler 480). For body fluid samples, miRNeasy Serum/Plasma Spike-In Control (*C. elegans* miR-39 miR mimic, QIAGEN) served as the control; while for cell samples, U6 snRNA (RNU6B, QIAGEN) served as the endogenous control. The relative levels of miRs were calculated by the 2^-*ΔΔ*Ct^ method [15].

### 2.7. Enzyme-Linked Immunosorbent Assay

Commercial ELISA-coated plates (Bioswamp, Wuhan, China) were used for ABCA1-labeled exosome capture. A total of 100 *μ*l of exosomal PBS solution was added to the reaction wells. After 30 minutes of incubation at 37°C, the plates were washed three times with PBS and drained; then, the RNA extraction reagent of the miRNeasy Serum/Plasma Kit (QIAGEN) was added to the wells, according to the kit instructions [16, 17]. The ABCA1 (Bioswamp) and CD9 (EXOAB-CD9A-1, System Biosciences) ELISA tests were performed in strict accordance with the instructions of the kit.

### 2.8. Statistical Analyses

Statistical analyses were performed using SPSS 18.0 for Windows (SPSS, Inc., Chicago, IL, USA). For normally distributed data, results are expressed as the mean ± standard deviation. The differences between groups were assessed using the one-way ANOVA. Rates were compared using the chi-square test. Correlations were determined by computing the Spearman Rank Correlation. *P* < 0.05 was considered to indicate a statistically significant difference.

## 3. Results

### 3.1. miR-193b Is Detected in Human RBC/WBC and RBC/WBC Medium, with Higher Levels in WBCs

**Figure 1:**
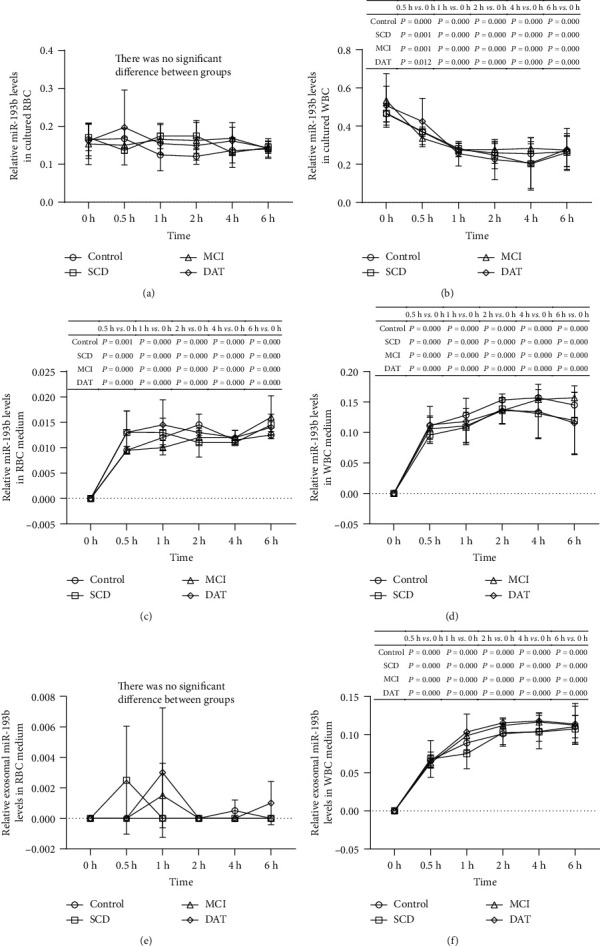
Total miR-193b and exosomal miR-193b detection in RBCs/WBCs and the medium of RBCs/WBCs. Relative miR-193b levels in cultured RBCs (a) and WBCs (b) at different points in time. Relative miR-193b levels in the medium of RBCs (c) and WBCs (d) at different points in time. Relative exosomal miR-193b levels in RBC (e) and WBC (f) culture medium at different points in time. There were 3 control, 3 SCD, 3 MCI, and 3 DAT samples for each time group.

To evaluate the potential utility of miR-193b as a noninvasive biomarker for AD in blood exosomes, we collected RBCs and WBCs from control subjects and patients with SCD, MCI, or DAT and analyzed the miR-193b distribution over a time course of cell culture. In RBCs, the total miR-193b levels were similar for all samples and were unchanged after 6 h of culture (*P* > 0.05) (Figure 1(a)); however, in WBCs, the total miR-193b levels were significantly decreased after 0.5 h in all groups (*P* < 0.05), with additional decrease at 1 h (Figure 1(b)). In contrast, the total secreted miR-193b levels in the RBC medium were significantly increased after 0.5 h of culture (*P* < 0.05) and then were stable thereafter in all groups (*P* > 0.05) (Figure 1(c)), while the total miR-193b levels in the WBC medium were significantly increased after 0.5 h of culture (*P* < 0.05) and then continued to increase slightly with peak levels at 2-4 h (Figure 1(d)). Notably, the absolute extracellular levels of miR-193b in the medium were much lower overall than the intracellular levels, especially for the RBCs (Figure 1(c)). These results suggest that miR-193b may reside in exosomes in WBCs.

To verify these findings, we directly evaluated the miR-193b levels in exosomes for both RBCs and WBCs. The exosomal levels in RBCs were low (Figure 1(e)); however, the exosomal miR-193b levels in WBCs showed a similar pattern as those in the medium, with significant increase after 0.5 h of culture (*P* < 0.05) (Figure 1(f)). Using these values at each time point, we calculated the average relative miR-193b level and miR-193b secretion ratio of RBCs and WBCs. The results based on data from all groups suggest that miR-193b is expressed in exosomes and that its overall expression is higher in WBCs than in RBCs (*P* < 0.05) (Table 2).

### 3.2. ABCA1 and ABCA1-Labeled Exosomes Are Detected at High Levels in Neurons

**Figure 2:**
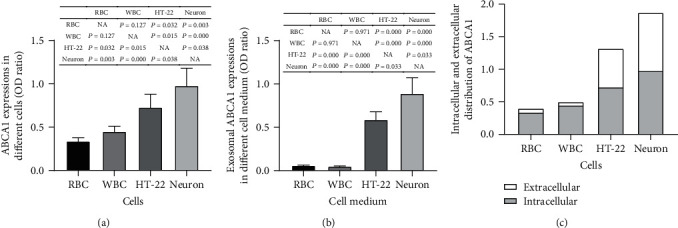
The ABCA1 and ABCA1-labeled exosome expression in RBCs, WBCs, HT-22 cells, and neurons: (a) ELISA results of ABCA1 expression in different cells; (b) ELISA results of exosomal ABCA1 levels in the medium of different cells; (c) the ratio of intracellular ABCA1 and extracellular ABCA1. For RBCs and WBCs, there were 3 control, 3 SCD, 3 MCI, and 3 DAT samples for each group; for HT-22 cells and neurons, there were 5 samples for each group.

Given the differences between miR-193b expression patterns in the intracellular and extracellular environments, we sought to evaluate the expression of the efflux mediator, ABCA1, in cultures of mouse cells. Our results demonstrate that ABCA1 is expressed in human RBCs, human WBCs, HT-22 mouse hippocampal neuron cells, and primary mouse neurons, with the highest expression in neurons (*P* < 0.05) (Figure 2(a)). The ABCA1 levels in exosomes harvested from HT-22 cells and the neuronal medium were also significantly higher than those of RBCs and WBCs (*P* < 0.05), with the highest exosomal ABCA1expression in the neuron group (*P* < 0.05) (Figure 2(b)). Furthermore, for HT-22 cells and neurons, nearly half (~40-50%) of the ABCA1 expression was detected in the extracellular medium, whereas for RBCs and WBCs, the ABCA1 expression was almost all (~80-90%) intracellular. These results suggest that HT-22 cells and primary neurons secrete relatively high levels of ABCA1-expressing exosomes.

### 3.3. ABCA1-Expressing Exosomes in CSF from AD Model APP1/PS1 Mice Can Be Evaluated In Vivo

**Figure 3:**
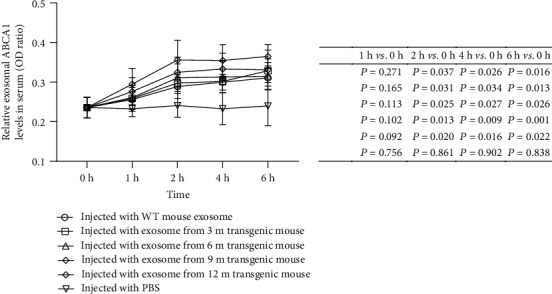
Levels of exosomal ABCA1 in the serum of WT mice at different points in time after injection of CSF exosomes from WT mice, APP/PS1 AD model mice of different ages, or PBS. After 2 h of injection, the serum exosomal ABCA1 levels of the CSF exosome injection groups were significantly higher than those of the 0-hour groups; the serum exosomal ABCA1 levels of the PBS group were significantly lower than those of the experimental groups.

To further evaluate the association of ABCA1 exosomal expression with AD in an in vivo system, we collected CFS from WT or APP1/PS1 mice of different ages and injected the CFS exosomes into wild-type mice. At 2 h after the injection, the exosomal ABCA1 levels in the serum were significantly higher for mice receiving APP/PS1-exosome injection than mice receiving WT-exosome injection (*P* < 0.05); this effect was somewhat age-dependent, with the highest expression in the 12 m APP/PS1-injection group (Figure 3, *n* = 10 mice per group). These results suggest that ABCA1-expressing exosomes from CFS can be evaluated in vivo.

### 3.4. ABCA1-Labeled Exosomal miR-193b Is Abundant in Serum and CSF from AD Model Mice

**Figure 4:**
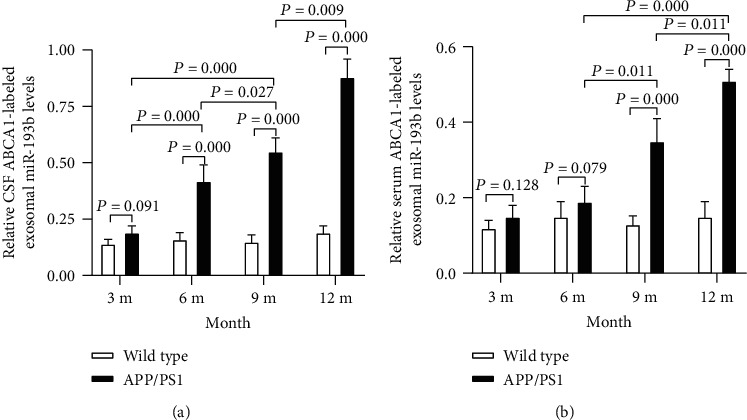
The ABCA1-labeled exosomal miR-193b levels in the serum and CSF of mice of different ages. The relative ABCA1-labeled exosomal miR-193b expression in CSF (a) and serum (b) of APP/PS1 transgenic mice and wild type mice was measured at different ages.

Using the transgenic mouse system, we evaluated the miR-193 expression in ABCA1-expressing exosomes. The ABCA1-labeled exosomal miR-193b levels were increased in the CSF of 6, 9, and 12 m APP/PS1 mice compared with WT mice, with the highest expression in the 12 m APP/PS1 mice (*P* < 0.05) (Figure 4(a)). Furthermore, the ABCA1-labeled exosomal miR-193b was also increased in the serum of 9 and 12 m APP/PS1 mice compared with WT mice, with the highest expression in the 12 m APP/PS1 mice (*P* < 0.05) (Figure 4(b)). These results provide in vivo evidence that miR-193b resides in ABCA1-labeled exosomes and suggests that it may be of higher abundance in AD model mice.

### 3.5. The Serum and CSF Exosomal ABCA1 Levels Are Elevated in AD Patients

**Figure 5:**
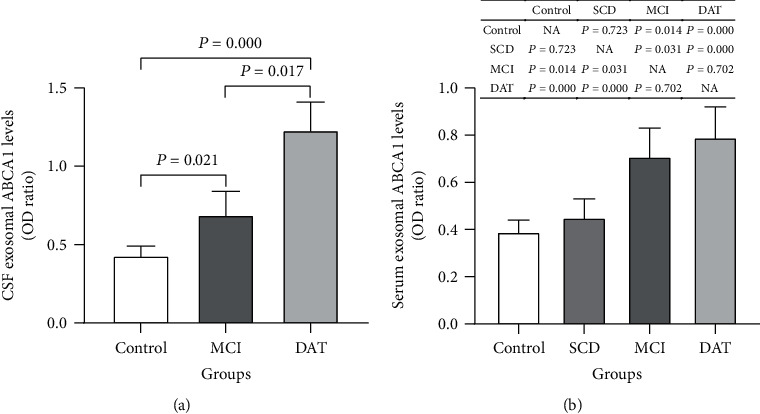
The serum and CSF exosomal ABCA1 levels in the SCD, MCI, DAT, and control groups. The exosomal ABCA1 expression in CSF of MCI, DAT, control groups (a) and serum of SCD, MCI, DAT, and control groups (b).

Given the predominate association of miR-193b with ABCA1-labeled exosomes in the APP/PS1 mouse model, we evaluated ABCA1 expression patterns in CSF and serum from AD patients. The exosomal ABCA1 expression was remarkably higher in both the CSF (Figure 5(a)) and the serum (Figure 5(b)) of MCI and DAT patients compared with the control group (*P* < 0.05). A slight increase of ABCA1 was also observed in the serum of SCD patients compared with the control group (*P* > 0.05) (Figure 5(b)). These results suggest that ABCA1-labeled exosomes may be more abundant in AD.

### 3.6. ABCA1-Labeled Exosomal miR-193b Levels Are Elevated in the CSF and Serum of AD Patients

**Figure 6:**
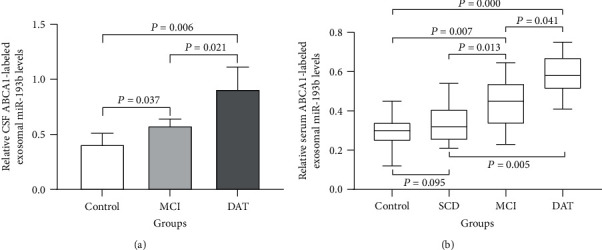
The relative ABCA1-labeled exosomal miR-193b levels in the serum and CSF of different groups. The relative ABCA1-labeled exosomal miR-193b levels in (a) CSF and (b) serum of MCI, DAT, and control groups and the SCD group (for (b)).

Given the higher levels of ABCA1-labeled exosomes in AD patients, we postulated that miR-193b-expressing ABCA1-labeled exosomes may also be elevated in AD. Indeed, our results demonstrate that the ABCA1-labeled exosomal miR-193b levels were increased in the CSF of the MCI and DAT patients compared with the control group, with the highest expression in DAT patients (*P* < 0.05) (Figure 6(a)). Furthermore, ABCA1-labeled exosomal miR-193b levels were slightly increased (*P* > 0.05) in the serum of SCD patients and significantly increased (*P* < 0.05) in the serum of MCI and DAT patients compared with the control group, with the highest expression in DAT patients (*P* < 0.05) (Figure 6(b)). Collectively, these results are consistent with a potential role for exosomal miR-193b as a biomarker of AD.

## 4. Discussion

AD is the fourth leading cause of death in the elderly population after cardiovascular disease, cerebrovascular disease, and cancer. As the population-aging trend has become increasingly apparent, AD has become more common in the elderly population. One of the characteristics of the disease is that the onset precedes the presentation. Neuronal cells may begin to show pathophysiological changes 10 to 20 years before diagnosis. At the same time, there is no effective curative treatment for patients entering the DAT phase; after the disease progresses, only symptomatic treatment is available to improve the quality of life [17, 18]. For patients with MCI, strategies have been developed to delay or prevent progression to the DAT phase by combining drugs with physical therapy.

Most early markers for disease diagnosis are directly or indirectly involved in the occurrence and development of disease-related symptoms, and the identification of candidate markers for early diagnosis of AD has been a priority in research endeavors [19]. Therefore, in this study, we used traditional methods to assess the potential value of miR-193b expression in the diagnosis of AD patients. Some miRs can be selectively packaged into exosomes and actively secreted, and the microvesicle-mediated secretion pathway is known to cross the blood-brain barrier [11, 20]. Therefore, we paired miR-193b detection with exosome capture using ABCA1 labeling. There are many sources of exosomes in the body, and we determined that in addition to the exosomal miR-193b production by neurons, WBCs can produce exosomes containing miR-193b, though the number of exosomes containing miR-193b in RBC cultures appears low, which is consistent with the results of other researchers' high-throughput studies [21]. The low miR-193b content in exosomes from WBCs could potentially cause false negative results in total exosomal miR-193b detection in some AD patients. Thus, in this study, we separated the exosomes in a timely manner (within half an hour) after collection to minimize interference caused by blood cell exosomes.

In recent years, a variety of specific exosome capture techniques have been developed, such as microfluidic chip technology and immune capture [16, 17]. The core principle of the immune capture method is based on the size of the exosomes (30-200 nm), which is comparable to the size of virus-like particles and viruses (within 300 nm) that can be captured by antibodies [22]. ABCA1 is a membrane protein that mediates the efflux of phospholipids and cholesterol to the extracellular receptor apolipoprotein A-I to generate new high-density lipoproteins [23]. Previous studies have shown that lipid and protein transport can accompany the transport of miRs in exosomes, so ABCA1 may also actively or passively transport miR-193b and other miRs during lipid transport [24, 25]. In this study, we demonstrated that WBCs, RBCs, HT-22 cells, and primary cultured neuronal cells each express ABCA1, but only HT-22 and primary neuronal cells released exosomes with high ABCA1 expression, which indicates that evaluation of ABCA1-labeled exosomes may provide an approach to eliminate the interference of low miR-193b exosomes released by WBC and RBC, thereby increasing the specificity of AD diagnosis.

Previous studies have predicted that exosomes can be used as carriers of information to facilitate paracrine transmission between cells [7, 18]. However, the blood-brain barrier is presumed to deter transmission of nucleic acids. In this study, high miR-193b exosomes extracted from the CSF of AD model mice were injected into the ventricles of WT mice to observe whether the appearance of high miR-193b exosomes could be transmitted across the blood-brain barrier to peripheral blood and be effectively detected. The results show that high miR-193b exosomes injected into the ventricle can increase the miR-193b in CSF and serum exosomes in wild-type mice, thus providing additional evidence for the passage of high miR-193b exosomes across the blood-brain barrier. APP expression is often abnormally elevated in AD, which is one of the reasons for the increased production of A*β* [19]. In a previous study, we identified miR-193b as a suppressor of APP [11]. miR-193b is downregulated in hippocampal neurons of APP/PS1 transgenic mice, but total exosomal miR-193b is upregulated [11, 12]. In the present study, we found that when the overall miR-193b levels decrease, the miR-193b under a certain component (the ABCA1-labeled exosome) tends to increase. Thus, the observed differences in miR-193b expression in AD may be an effort made by the body to increase miR-193b to compensate for APP loss. However, the precise biological roles of miR-193b in AD and whether the signal transmitted through the APCA1-labeled exosome can further exert functional effects remain to be further studied.

Importantly, our clinical research results show that ABCA1 levels are higher in AD. Furthermore, the sensitivity of miR-193b in AD diagnosis was greatly improved after the use of ABCA1-labeled exosome capture technology to detect specific exosomes. Though we have attempted to better distinguish total exosomal miR-193b levels in patients using a higher cut-off value, it caused higher false negative rates. Therefore, although the detection process is more complicated than the direct extraction of total exosomes, the isolation of ABCA1-labeled exosomes is essential for high specificity AD diagnosis. In laboratories that use automatic ELISA sample loading and plate washing machines, these operations will not consume much extra labor and will greatly improve diagnosis. Given that ABCA1-labeled exosomes were upregulated in SCD subjects, this approach may be helpful for early diagnosis of AD, which is otherwise difficult to diagnose.

In summary, this study provides a method to capture ABCA1-labeled exosomes and determine the associated miR-193b levels, which provides a more efficient approach for early diagnosis of AD. The use of specific exosomal surface markers for differentiation and identification will identify the source of exosomes more effectively and accurately and ultimately improve the detection and diagnosis for clinical AD patients.

## Figures and Tables

**Table 1:** Demographics and clinical characteristics of subjects in this study.

Variable	Control (serum)	Control (CSF)	SCD (serum)	MCI (serum)	MCI (CSF)	DAT (serum)	DAT (CSF)
No. of subjects	60	6	89	92	16	92	11
Age (years)	76.5 ± 6.1	69.4 ± 3.3	75.7 ± 4.9	75.2 ± 8.0	68.2 ± 5.1	78.1 ± 7.2	72.1 ± 4.3
Gender (% males)	50.0	57.1	54.5	55.9	57.9	55.0	53.8
Creatinine (mg/dl)	0.85 ± 0.14	0.77 ± 0.18	0.78 ± 0.20	0.76 ± 0.35	0.73 ± 0.33	0.81 ± 0.27	0.83 ± 0.15
Homocysteine (*μ*mol/l)	8.5 ± 2.1	8.3 ± 1.7	8.5 ± 5.7	16.8 ± 5.1	18.2 ± 2.2	18.2 ± 5.3	21.2 ± 6.3
Body mass index (BMI, kg/m^2^)	25.5 ± 2.7	26.8 ± 3.5	26.3 ± 3.0	26.3 ± 2.8	26.2 ± 3.2	27.5 ± 4.1	25.2 ± 2.5
Heart rate (per min)	75.3 ± 8.3	75.7 ± 5.4	76.3 ± 7.5	73.5 ± 11.4	71.7 ± 7.2	75.6 ± 10.5	76.2 ± 9.7
Systolic blood pressure (mmHg)	122.0 ± 11.3	128.5 ± 7.9	121 ± 13.8	125.2 ± 16.9	122.7 ± 11.3	132.0 ± 16.2	127.0 ± 12.0
Diastolic blood pressure (mmHg)	78.9 ± 12.2	81.5 ± 9.1	83.2 ± 13.1	86.2 ± 15.9	87.2 ± 11.0	87.0 ± 15.9	83.5 ± 13.0
Hypertension treatment (%)	16.7	23.5	13.9	13.3	10.5	16.3	15.4
Statin treatment (%)	16.7	23.5	15.2	15.4	15.8	20.8	30.8

**Table 2:** The average miR-193b secretion ratio of cultured RBC and WBC.

Cell type	Total relative miR-193b levels	Total miR-193b secretion ratio		Exosomal miR-193b secretion ratio	
		0.5 h	4 h	0.5 h	4 h
RBC	0.107	6.95%	7.29%	0.00%	0.01%
WBC	0.411^∗^ (*P* = 0.003)	25.77%^∗^ (*P* = 0.000)	26.57%^∗^ (*P* = 0.000)	11.13%^∗^ (*P* = 0.000)	18.55%^∗^ (*P* = 0.000)

∗There were significant differences compared with the RBC group (*P* < 0.05), *n* = 12.

## Data Availability

The data used to support the findings of this study are available from the first author or corresponding author upon request.
